# Trait-specific consequences of inbreeding on adaptive phenotypic plasticity

**DOI:** 10.1002/ece3.1339

**Published:** 2014-12-03

**Authors:** Mads F Schou, Torsten N Kristensen, Volker Loeschcke

**Affiliations:** 1 Department of Bioscience, Aarhus UniversityAarhus C, DK-8000, Denmark; 2 Department of Chemistry and Life Science, Aalborg UniversityAalborg East, DK-9220, Denmark

**Keywords:** Developmental plasticity, inbreeding–environment interactions, insects, pigmentation, temperature, wing size and shape

## Abstract

Environmental changes may stress organisms and stimulate an adaptive phenotypic response. Effects of inbreeding often interact with the environment and can decrease fitness of inbred individuals exposed to stress more so than that of outbred individuals. Such an interaction may stem from a reduced ability of inbred individuals to respond plastically to environmental stress; however, this hypothesis has rarely been tested. In this study, we mimicked the genetic constitution of natural inbred populations by rearing replicate *Drosophila melanogaster* populations for 25 generations at a reduced population size (10 individuals). The replicate inbred populations, as well as control populations reared at a population size of 500, were exposed to a benign developmental temperature and two developmental temperatures at the lower and upper margins of their viable range. Flies developed at the three temperatures were assessed for traits known to vary across temperatures, namely abdominal pigmentation, wing size, and wing shape. We found no significant difference in phenotypic plasticity in pigmentation or in wing size between inbred and control populations, but a significantly higher plasticity in wing shape across temperatures in inbred compared to control populations. Given that the norms of reaction for the noninbred control populations are adaptive, we conclude that a reduced ability to induce an adaptive phenotypic response to temperature changes is not a general consequence of inbreeding and thus not a general explanation of inbreeding–environment interaction effects on fitness components.

## Introduction

The ability of organisms to adapt to climatic stressors, such as high temperatures, through plastic or evolutionary responses, has received much attention lately (Gienapp et al. 2008; Sunday et al. 2011; Diamond et al. 2012; Zeuss et al. 2014). Effects of environmental stress, and thereby the need to adapt, may be more severe in inbred populations due to synergistic inbreeding–environment interactions (Fox and Reed 2011; Reed et al. 2012). In novel or changing environments, such interactions may decrease the fitness of inbred individuals more compared to outbred individuals. Little is known about the causation of the inbreeding–stress interactions, although they may determine the efficiency of selection and persistence of small natural populations in harsh environments. Inbreeding–environment interactions may stem from a decreased ability of inbred individuals to induce adaptive phenotypic plasticity (Bijlsma and Loeschcke 2011; Reed et al. 2012), but only few studies have addressed this possibility. Adaptive phenotypic plasticity for shell thickness, as a response to predator presence or absence, decreased with inbreeding in a freshwater snail (Auld and Relyea 2010). Conversely, inbreeding did not affect the ability to increase cold resistance in response to developmental cold acclimation across multiple *Drosophila* species (Kristensen et al. 2011).

In this study, we investigated the effects of inbreeding on adaptive phenotypic plasticity of abdominal pigmentation and wing size and shape across a developmental thermal gradient in *D. melanogaster*. We have chosen these traits not only because they show a strong phenotypic change across developmental temperatures (Gibert et al. 2004), but also because the plasticity has been shown to be adaptive: A darker abdomen is favorable in colder environments, while a pale abdomen is so in hot environments (Kingsolver 1995; Clusella-Trullas et al. 2008; Matute and Harris 2013), and wing size and shape change with temperature partly in order to optimize takeoff and flight abilities (Azevedo et al. 1998; Hoffmann et al. 2007; Frazier et al. 2008). Given that the norms of reaction across temperatures of outbred individuals are adaptive for the traits investigated, any directional change (decrease or increase) in phenotypic plasticity will by definition result in a reduced adaptive phenotypic plasticity and in a fitness reduction. Thus, a reduced adaptive phenotypic plasticity may not only arise from a decreased ability of inbred individuals to respond to environmental change as often described in the literature (Bijlsma and Loeschcke 2005; Reed et al. 2012), but also from an increased phenotypic plasticity of inbred compared to outbred individuals. An increase in phenotypic plasticity can be due to an increased environmental sensitivity, that is, inbred individuals have a lower stress threshold compared to outbred individuals.

## Materials and Methods

Prior to the creation of replicate inbred populations, we established a *D. melanogaster* population from 589 wild females caught in Denmark; for details see Schou et al. (2014). The population was genetically diverse, with nucleotide diversity estimates in the range reported for multiple *D. melanogaster* populations (*π *= 0.48%) (Pool et al. 2012; Schou et al. 2014). To mimic the process of genetic drift and selection (purging) in small natural populations, we reared each of ten replicate populations at a population size of 10 (N10). As a contrast to the replicate N10 populations, three replicate populations were established and reared at a population size of 500 (control). We controlled rearing density during development and maintained a 1:1 sex ratio, while the diurnal light and temperature (mean: 24.8°C) cycle followed a Gaussian curve mimicking a natural diurnal cycle; for rearing details see Jensen et al. (2014). After 25 generations, we expanded the population size of the replicate N10 populations to 250 for 7 generations before initiating the experiment. The expected inbreeding coefficient (*F*) of replicate N10 populations was estimated to 0.84 (Crow and Kimura 1970) by assuming *f*_0_ = 0 and *Ne* = 2/3 N (Buri 1956; Nunney 1993 [lottery polygyny and Poison distributed offspring per mating]).

From each of the 13 replicate populations, we transferred 16 vials with 40 eggs to three different constant thermal regimes: cold (14°C), benign (25°C), and warm (31°C). Emerging adults were kept at their respective developmental temperatures until 2 days of age to complete the melanisation (Chakir et al. 2002). At this point, flies were transferred to a 1:1:8 glycerol/acetic acid/ethanol mixture. We assessed the pigmentation of abdominal segments 2 to 7 of ∼20 females per replicate population in each regime (Fig. S1). We focused on females throughout this study as they show a larger absolute plasticity in abdominal pigmentation, as opposed to males in which the anterior abdominal segments remain dark across developmental temperatures (Gibert et al. 2009). Each tergite was assigned a score between 0 and 10, with 0: No part of the tergite was black, and 10: The entire tergite was black (David et al. 1990). To calculate the total pigmentation of each fly, we weighted each tergite by its area; values 1, 0.75, and 0.26 for tergites 5, 6, and 7, respectively (Petavy et al. 2002), while tergites 2–4 were weighted with value 1.

We measured the right wing (right when positioned on the abdomen) on ∼23 flies per replicate population per thermal regime. Wings were mounted on glass slides in an alcohol/glycerine solution. Images of the individual wings were obtained with a Leica MZ 125 microscope, a Leica DFC295 camera, and the software Leica Application Suite 3.7.0 (Leica Microsystems, Wetzlar, Germany). As an estimate of wing size, we calculated the centroid size based on 11 landmarks (Trotta et al. 2010). We further estimated wing shape by calculating the ratio between wing length (distance from landmark 3 to 6) and width (distance from landmark 2 to 4) (Fig. S2).

We analyzed the effect of inbreeding on the adaptive phenotypic plasticity of total pigmentation (square root transformed), wing size and shape using general linear mixed models (glmm) (Bates et al. 2014). Differences in the phenotypic change across temperatures between replicate N10 and control populations would show up as a significant interaction between the two fixed effects *breeding regime* (control or N10) and *temperature* (Schlichting 1986; Valladares et al. 2006) and will be interpreted as evidence for a reduced adaptive phenotypic plasticity in inbred individuals. *Temperature* was modelled as a quadratic continuous term to allow possible differences in the curvature of the norm of reaction between breeding regimes. We included random intercept and slopes of replicate populations across temperatures in the model. *P*-values were obtained by sequential model reduction and by model comparisons with likelihood ratio tests. In case of a significant interaction, no further model reductions were performed. All statistical analyses were performed in R (R Core Team, 2014)

## Results

**Figure 1:**
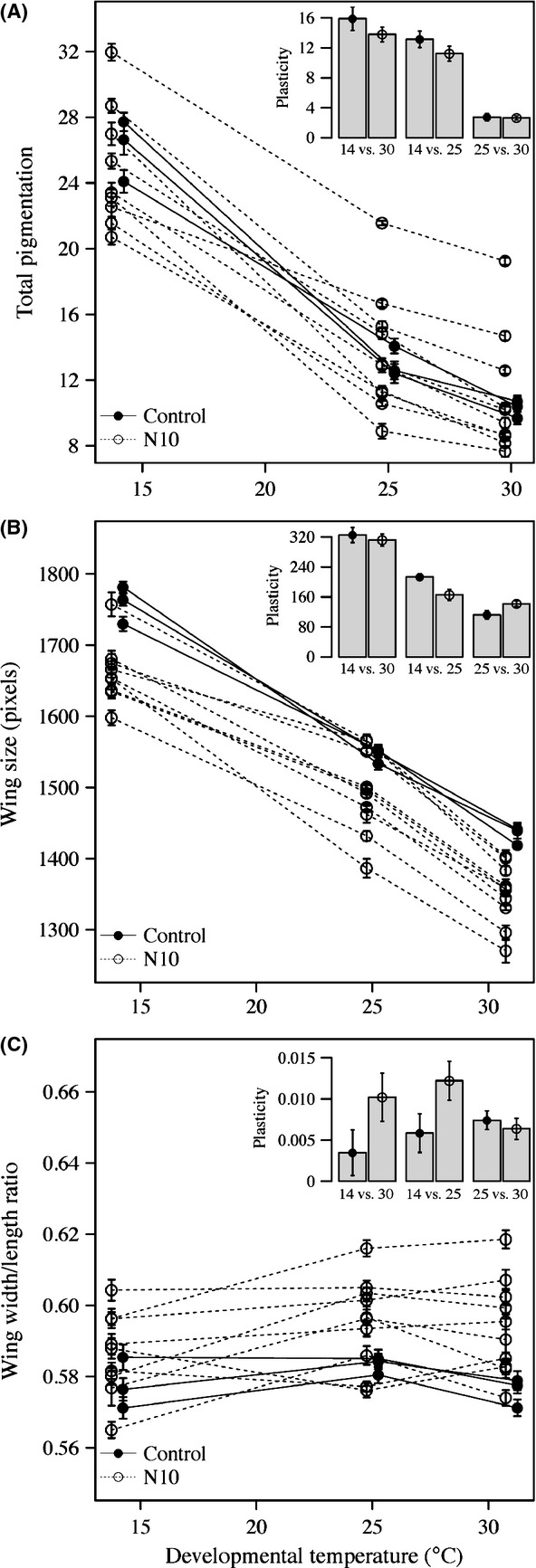
Norm of reaction for total pigmentation (A), wing size (B), and wing shape (C) of replicate N10 (*n* = 10) and control (*n* = 3) populations. In each plot, the plasticity (absolute change in phenotype) for the three contrasts is summarized in a barplot. Error bars are standard errors.

Total pigmentation decreased by 57% as the temperature was increased from 14 to 31°C (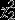 = 43.42, *P < *0.001), but was not affected by *breeding regime* (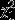 = 0.002, *P = *0.97) or by the interaction between *breeding regime* and *temperature* (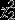 = 0.92, *P = *0.63), although one replicate N10 population showed a strongly decreased response in pigmentation to temperature changes (Fig.1A; Figs S3 and S4). Wing size decreased by 19% from 14 to 31°C (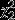 = 61.47, *P < *0.001) and was affected by *breeding regime* (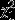 = 7.63, *P < *0.01) such that replicate N10 populations on average had wings that were 6% smaller than control populations (Fig.1B). However, as for total pigmentation, there was no effect of the interaction between *temperature* and *breeding regime* on wing size (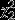 = 4.27, *P = *0.12). The interaction between *temperature* and *breeding regime* was significant for wing shape (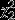 = 6.44, *P *<* *0.05; Fig.1C), showing a small but significantly higher rate of change in wing shape across temperatures in replicate N10 (an increase of 2% from 14 to 31°C) compared to control populations in which no increase was detected. The significant interaction was primarily driven by a difference in the overall curvature between the two breeding regimes (Fig.1C).

## Discussion

Here, we explore how inbreeding may affect the adaptive phenotypic plasticity of three traits using replicate inbred and outbred populations. There is a strong experimental evidence that the norms of reaction of the traits investigated are adaptive (Kingsolver 1995; Azevedo et al. 1998; Hoffmann et al. 2007; Clusella-Trullas et al. 2008; Frazier et al. 2008; Matute and Harris 2013); therefore, we find it appropriate to interpret any differentiation in the norm of reaction of the replicate N10 populations from the control populations as evidence for a decreased adaptive phenotypic plasticity. We found no difference in phenotypic plasticity between control and N10 populations in neither total pigmentation nor wing size and thus no evidence of a reduced adaptive phenotypic plasticity with inbreeding for these traits. However, inbreeding did significantly reduce wing size across temperatures, which may reduce flight ability in inbred flies (Frazier et al. 2008). The wing shape of replicate N10 populations showed a significant higher phenotypic plasticity compared to replicate control populations (Fig.1C). We interpret this as a reduced adaptive phenotypic plasticity in this trait. Our results do not support the hypothesis that a reduced adaptive phenotypic plasticity is a general consequence of inbreeding; instead, such an effect seems to be trait and population specific.

Phenotypic plasticity is triggered by environmental cues followed by a phenotypic change. A reduced ability to adjust the phenotype should be distinguished from a changed environmental sensitivity (i.e., a changed ability to detect a cue or a changed response threshold). For example, *Drosophila* studies suggest that inbreeding increases the environmental sensitivity by lowering the temperature at which the heat shock protein Hsp70 is upregulated in larvae (Kristensen et al. 2002; Pedersen et al. 2005). Conversely, disruption of neural circuits controlling behavioral strategies (Garrity et al. 2010) may decrease environmental sensitivity. As such, the inconsistency in the consequences of inbreeding on phenotypic changes across environments observed here and elsewhere (Auld and Relyea 2010; Kristensen et al. 2011) may be a result of unpredictable net outcomes of inbreeding effects on environmental sensitivity.

Inbreeding can result in increased energy expenditure for maintenance metabolism (Koehn and Bayne 1989; Myrand et al. 2002; Parsons 2005); thus, it is relevant whether the plastic response investigated is energetically costly. It is largely accepted that phenotypic changes during development and at the adult stage are constituted by different mechanisms (Angiletta 2009). During development, the organism can adjust the phenotype of a given trait while the trait is being developed. Conversely, a change in the phenotype of the trait at the adult stage requires changes made to a fully developed trait. We speculate that a phenotypic change during development may be less expensive than a phenotypic change at the adult stage. While Auld and Relyea (2010) found a decreased adaptive phenotypic plasticity in shell thickness as a response to predator presence or absence in 29-day-old snails, we studied responses that can be classified as developmental adaptive phenotypic plasticity (i.e., the phenotypic change takes place during the development of the organism into the adult stage). The difference in the timing of the phenotypic change may explain the discrepancy between studies (Auld and Relyea 2010; Kristensen et al. 2011).

The current study does not allow for a mechanistic distinction between inbreeding effects on the capacity to adjust the phenotype and environmental sensitivity (i.e., inbred individuals have a lower stress threshold). Future studies should consider this if we are to understand the inconsistency in the results obtained within research on inbreeding by environment interactions and more generally genotype by environment interactions. In conclusion, a lower adaptive phenotypic plasticity is not a general consequence of inbreeding or a general explanation for inbreeding–environment interactions.

## Data Accessibility

Data will be archived in an appropriate database such as Dryad if the manuscript is finally accepted. Data identifier: doi:10.5061/dryad.ts56p.

## Supporting Information

**Figure S1.**Pigmentation assessment. **Figure S2.** Wing landmarks. **Figure S3.** Tergite and replicate population specific pigmentation. **Figure S4.** Tergite specific pigmentation means.

## References

[b1] Angiletta MJ (2009). Thermal adaptation: a theoretical and empirical analysis.

[b2] Auld JR, Relyea RA (2010). Inbreeding depression in adaptive plasticity under predation risk in a freshwater snail. Biol. Lett.

[b3] Azevedo RBR, James AC, McCabe J, Partridge L (1998). Latitudinal variation of wing: thorax size ratio and wing-aspect ratio in *Drosophila melanogaster*. Evolution.

[b4] Bates D, Maechler M, Bolker B, Walker S (2014). Lme4: linear mixed-effects models using Eigen and S4. R package version 1.1–5.

[b5] Bijlsma R, Loeschcke V (2005). Environmental stress, adaptation and evolution: an overview. J. Evol. Biol.

[b6] Bijlsma R, Loeschcke V (2011). Genetic erosion impedes adaptive responses to stressful environments. Evol. Appl.

[b7] Buri P (1956). Gene frequency in small populations of mutant *Drosophila*. Evolution.

[b8] Chakir M, Chafik A, Gibert P, David JR (2002). Phenotypic plasticity of adult size and pigmentation in *Drosophila*: thermosensitive periods during development in two sibling species. J. Therm. Biol.

[b9] Clusella-Trullas S, Terblanche JS, Blackburn TM, Chown SL (2008). Testing the thermal melanism hypothesis: a macrophysiological approach. Funct. Ecol.

[b10] Crow JF, Kimura M (1970). An introduction to population genetics theory.

[b11] David JR, Capy P, Gauthier JP (1990). Abdominal pigmentation and growth temperature in *Drosophila melanogaster*: similarities and differences in the norms of reaction of successive segments. J. Evol. Biol.

[b12] Diamond SE, Sorger DM, Hulcr J, Pelini SL, Del Toro I, Hirsch C (2012). Who likes it hot? A global analysis of the climatic, ecological, and evolutionary determinants of warming tolerance in ants. Glob. Chang. Biol.

[b13] Fox CW, Reed DH (2011). Inbreeding depression increases with environmental stress: an experimental study and meta-analysis. Evolution.

[b14] Frazier MR, Harrison JF, Kirkton SD, Roberts SP (2008). Cold rearing improves cold-flight performance in *Drosophila* via changes in wing morphology. J. Exp. Biol.

[b15] Garrity PA, Goodman MB, Samuel AD, Sengupta P (2010). Running hot and cold: behavioral strategies, neural circuits, and the molecular machinery for thermotaxis in *C. elegans* and *Drosophila*. Genes Dev.

[b16] Gibert P, Capy P, Imasheva A, Moreteau B, Morin JP, Petavy G (2004). Comparative analysis of morphological traits among *Drosophila melanogaster* and *D. simulans*: genetic variability, clines and phenotypic plasticity. Genetica.

[b17] Gibert P, Moreteau B, David JR (2009). Phenotypic plasticity of abdomen pigmentation in two geographic populations of *Drosophila melanogaster*: male-female comparison and sexual dimorphism. Genetica.

[b18] Gienapp P, Teplitsky C, Alho JS, Mills JA, Merilä J (2008). Climate change and evolution: disentangling environmental and genetic responses. Mol. Ecol.

[b19] Hoffmann AA, Ratna E, Sgrò CM, Barton M, Blacket M, Hallas R (2007). Antagonistic selection between adult thorax and wing size in field released *Drosophila melanogaster* independent of thermal conditions. J. Evol. Biol.

[b20] Jensen P, Overgaard J, Loeschcke V, Schou MF, Malte H, Kristensen TN (2014). Inbreeding effects on standard metabolic rate investigated at cold, benign and hot temperatures in *Drosophila melanogaster*. J. Insect Physiol.

[b21] Kingsolver JG (1995). Fitness consequences of seasonal polyphenism in western white butterflies. Evolution.

[b22] Koehn RK, Bayne BL (1989). Towards a physiological and genetical understanding of the energetics of the stress response. Biol. J. Linn. Soc.

[b23] Kristensen T, Dahlgaard J, Loeschcke V (2002). Inbreeding affects Hsp70 expression in two species of *Drosophila* even at benign temperatures. Evol. Ecol. Res.

[b24] Kristensen TN, Loeschcke V, Bilde T, Hoffmann AA, Sgró C, Noreikien≐ K (2011). No inbreeding depression for low temperature developmental acclimation across multiple *Drosophila* species. Evolution.

[b25] Matute DR, Harris A (2013). The influence of abdominal pigmentation on desiccation and ultraviolet resistance in two species of *Drosophila*. Evolution.

[b26] Myrand B, Tremblay R, Sévigny J-M (2002). Selection against blue mussels (*Mytilus edulis* L.) homozygotes under various stressful conditions. J. Hered.

[b27] Nunney L (1993). The influence of mating system and overlapping generations on effective population size. Evolution.

[b28] Parsons PA (2005). Environments and evolution: interactions between stress, resource inadequacy and energetic efficiency. Biol. Rev. Camb. Philos. Soc.

[b29] Pedersen KS, Kristensen TN, Loeschcke V (2005). Effects of inbreeding and rate of inbreeding in *Drosophila melanogaster*-Hsp70 expression and fitness. J. Evol. Biol.

[b30] Petavy G, Moreteau B, Gibert P, David JR, Pétavy G (2002). Phenotypic plasticity of body pigmentation in *Drosophila*: influence of a developmental thermoperiodic regime in two sibling species. Physiol. Entomol.

[b31] Pool JE, Corbett-Detig RB, Sugino RP, Stevens KA, Cardeno CM, Crepeau MW (2012). Population Genomics of sub-saharan *Drosophila melanogaster*: African diversity and non-African admixture. PLoS Genet.

[b32] Reed DH, Fox CW, Enders LS, Kristensen TN (2012). Inbreeding-stress interactions: evolutionary and conservation consequences. Ann. N. Y. Acad. Sci.

[b39] R Core Team (2014).

[b33] Schlichting CD (1986). The evolution of phenotypic plasticity in plants. Annu. Rev. Ecol. Syst.

[b34] Schou MF, Kristensen TN, Kellermann V, Schlötterer C, Loeschcke V (2014). A *Drosophila* laboratory evolution experiment points to low evolutionary potential under increased temperatures likely to be experienced in the future. J. Evol. Biol.

[b35] Sunday JM, Bates AE, Dulvy NK (2011). Global analysis of thermal tolerance and latitude in ectotherms. Proc. R. Soc. Lond. B Biol. Sci.

[b36] Trotta V, Pertoldi C, Rudoy A, Manenti T, Cavicchi S, Guerra D (2010). Thermal plasticity of wing size and shape in *Drosophila melanogaster**D. simulans* and their hybrids. Clim. Res.

[b37] Valladares F, Sanchez-Gomez D, Zavala MA (2006). Quantitative estimation of phenotypic plasticity: bridging the gap between the evolutionary concept and its ecological applications. J. Ecol.

[b38] Zeuss D, Brandl R, Brändle M, Rahbek C, Brunzel S (2014). Global warming favours light-coloured insects in Europe. Nat. Commun.

