# Supplementary material for: Trait-specific consequences of inbreeding on adaptive phenotypic plasticity
Source: Ecol Evol. 2014 Dec 3;5(1):1–6. doi: 10.1002/ece3.1339 (PMC4298428; doi:10.1002/ece3.1339)
Supplement: Supplementary file 1 — Figure S1.Pigmentation assessment. Figure S2. Wing landmarks. Figure S3. Tergite and replicate population specific pigmentation. Figure S4. Tergite specific pigmentation means. [file ece30005-0001-sd1.docx]

Fig. S1 Pigmentation assessment. The average abdominal pigmentation of a sample (a replicate population from a given temperature) was obtained by independent and randomized estimation of pigmentation for two groups of ten flies. This figure illustrates the correlation between the average total pigmentation from the two groups within each sample.

Fig. S2 Wing landmarks. The 11 landmarks on the wing used to estimate wing size, from the centroid size, and to estimate wing shape, as the ratio between wing length (distance from landmark 3 to 6) and width (distance from landmark 2 to 4).


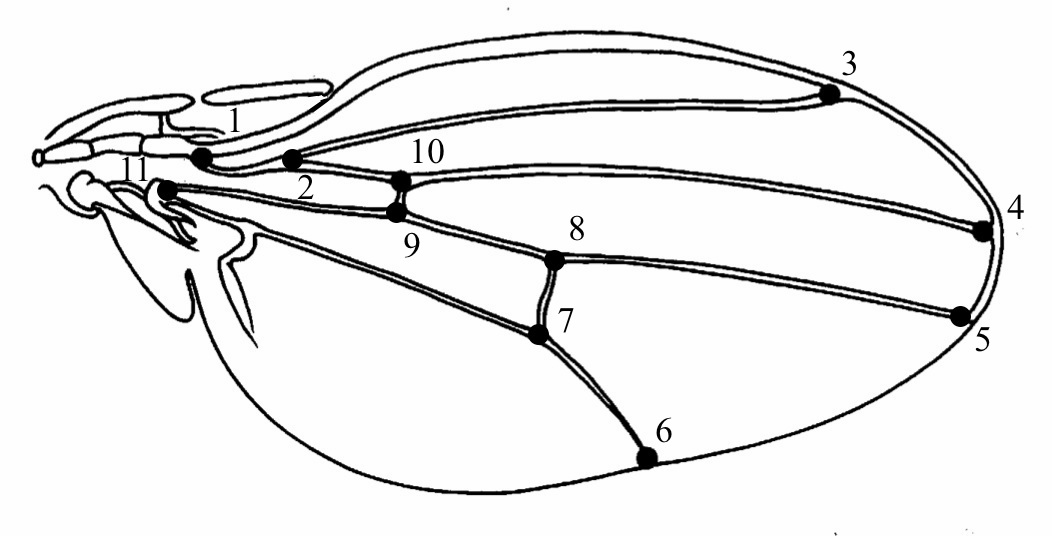


Fig. S3 Tergite and replicate population specific pigmentation. Norms of reaction for pigmentation for each of the replicate N10 and Control populations. Each graph represents one of the six measured tergites. Error bars are standard errors.

Fig. S4 Tergite specific pigmentation means. Norms of reaction for each breeding regime. Each graph represents one of the six measured tergites. Error bars are standard errors.
